# Draft Genome Sequence of Clostridium beijerinckii Strain mbf-VZ-132, Isolated from an Environmental Soil Sample

**DOI:** 10.1128/MRA.00131-21

**Published:** 2021-04-29

**Authors:** Nils Thieme, Wolfgang Liebl, Vladimir V. Zverlov

**Affiliations:** a Chair of Microbiology, TUM School of Life Sciences Weihenstephan, Technical University of Munich, Freising, Germany; b Institute of Molecular Genetics, National Research Centre, Kurchatov Institute, Moscow, Russia; University of Arizona

## Abstract

Clostridium beijerinckii strain mbf-VZ-132 was isolated from soil in Freising-Weihenstephan (Bavaria, Germany). The 16S rRNA gene sequence showed a 99.9% sequence identity to that of Clostridium diolis DSM 15410, which was recently reclassified as C. beijerinckii. In this study, we present the draft genome sequence of C. beijerinckii mbf-VZ-132 based on PacBio sequencing.

## Abstract (precis)

Clostridium beijerinckii strain mbf-VZ-132 was isolated from soil in Freising-Weihenstephan (Bavaria, Germany). The 16S rRNA gene sequence showed a 99.9% sequence identity to Clostridium diolis DSM 15410, which was recently reclassified as C. beijerinckii. In this study, we present the draft genome sequence of C. beijerinckii mbf-VZ-132, based on PacBio sequencing.

## ANNOUNCEMENT

*Clostridium* spp. (phylum *Firmicutes*, order *Clostridiales*, family *Clostridiaceae*) are mostly anaerobic, Gram-positive, endospore-forming soil bacteria (1). Some solventogenic *Clostridia* strains are able to utilize polysaccharides from plant biomass for acetone-butanol-ethanol (ABE) fermentation (2, 3). Hence, we screened the strain collection gathered at the Chair of Microbiology (Technical University of Munich, Germany) for *Clostridia* strains that were able to perform ABE fermentation on milling by-products (2). One of the strains, mbf-VZ-132, produced above-average butanol yields on wheat red dog. Wheat red dog is a by-product of the wheat milling process produced year round that is rich in starch and protein and commonly used as animal feed (4). The strain was incubated for 72 h at 34°C in 50 ml YAF25 liquid Grundmedium (GM) (2) with 15% (wt/vol) wheat red dog as the carbon source. The fermentation products (acetate, butyrate, acetone, 1-butanol, and ethanol) were measured by gas chromatography on a Nexis gas chromatography (GC) 2030 system (Shimadazu, Kyoto, Japan), as described previously (2). Strain mbf-VZ-132 produced ∼14.4 g/liter 1-butanol (Table 1). These experiments were performed in biological triplicate.

**TABLE 1:** Fermentation products of Clostridium beijerinckii strain mbf-VZ-132^*a*^

Product	Amt (g/liter)
Acetate	3.16 ± 0.98
Butyrate	4.06 ± 1.24
Acetone	1.77 ± 0.15
1-Butanol	14.37 ± 0.91
Ethanol	ND^*b*^

a The strain was incubated for 72 h at 34°C in 50 ml GM with 15% (wt/vol) wheat red dog as the carbon source. The sample size was three biological replicates.

b ND, not detected.

Strain mbf-VZ-132 was collected from a soil sample recovered in Freising-Weihenstephan (Bavaria, Germany) in April 2011. An enrichment culture was generated by applying the soil to a potato with sterilized toothpicks. The potato was incubated in tap water for 72 h at 35°C. A dilution series from 1 × 10^−2^ to 1 × 10^−8^ was performed in 1× phosphate-buffered saline (PBS) and spread onto clostridial growth medium (CGM) (5) plus 1.8% (wt/vol) agar (Carl Roth, Karlsruhe, Germany) plates. After 24 h of incubation at 35°C, colonies were picked and incubated in liquid CGM. The clonal purity and species were determined by amplifying and sequencing the 16S rRNA gene of mbf-VZ-132 with the primers 27F (AGAGTTTGATCMTGGCTCAG) and 1492R (CGGTTACCTTGTTACGACTT). The sequence obtained had a similarity of 99.9% to that of Clostridium diolis DSM 15410, which was recently reclassified as Clostridium beijerinckii (6, 7). To analyze C. beijerinckii mbf-VZ-132, its genomic DNA was sequenced using PacBio sequencing.

C. beijerinckii mbf-VZ-132 chromosomal DNA was extracted from a clonal pure culture, which was cultivated anaerobically in liquid GM YAF25 with 4% (wt/vol) glucose (Carl Roth) as the carbon source at 34°C for 48 h. The DNA was extracted using the MagAttract high-molecular-weight (HMW) DNA kit from Qiagen (Hilden, Germany) and sent to Novogene Co., Ltd. (Bejing, China), for PacBio sequencing. The chromosomal DNA was analyzed using a Qubit fluorometer (Thermo Fisher Scientific, Waltham, MA, USA). After quality confirmation, DNA was randomly sheared to create SMRTbell libraries. Hairpin adaptors were ligated to both ends of double-stranded DNA (dsDNA) to create SMRTbell templates. Hairpin dimers were removed using a MagBead kit (Pacific Biosciences, CA, USA). Finally, failed ligation products were removed with exonucleases and AMPure PB beads (Pacific Biosciences). Sequencing primers were annealed to the SMRTbell templates, and they were loaded onto a single-molecule real-time (SMRT) cell. The SMRT cell was sequenced using a PacBio RS II system. A total of 61,436 reads were generated, with a mean length of 8,365 bp and an *N*_50_ value of 10,758 bp.

Raw data filtering was conducted using the Novogene in-house pipeline. Briefly, sequences with a size of <500 bp were filtered out, followed by an initial assembly. The obtained sequences of the initial assembly were aligned with the raw BAM file to acquire a new clean BAM file. The assembly was performed using SMRT Link V2.3.0 with default parameters. The assembly resulted in 4 contigs with 80.0× coverage. The contigs had a combined length of 6,088,663 bp, a G+C content of 29.9 mol%, and an *N*_50_ value of 5,288,266 bp. Totals of 5,155 coding sequences and 154 RNAs (17 5S rRNAs, 16 16S rRNAs, 16 23S rRNAs, 99 tRNAs, and 6 noncoding RNAs [ncRNAs]) were identified using the NCBI Prokaryotic Genome Annotation Pipeline for prediction and annotation of open reading frames (ORFs) (8).

The genome sequence of C. beijerinckii mbf-VZ-132 presented here will be of value for future examination of the biotechnological potential of this strain. Furthermore, it will enhance our ability to optimize this strain for ABE fermentation of plant biomass-based waste and side streams.

### Data availability.

This whole-genome shotgun sequencing project has been deposited at DDBJ/ENA/GenBank under the accession number JADOEF000000000. The version described in this paper is version JADOEF010000000. The raw sequencing reads are provided in the Sequence Read Archive (SRA) under the accession number SRR13071105. The BioProject entry with the accession number PRJNA674814 summarizes the whole project in NCBI and contains one BioSample entry with the accession number SAMN16675373.
